# Heterochronic Parabiosis Causes Dacryoadenitis in Young Lacrimal Glands

**DOI:** 10.3390/ijms24054897

**Published:** 2023-03-03

**Authors:** Kaitlin K. Scholand, Alexis F. Mack, Gary U. Guzman, Michael E. Maniskas, Ritu Sampige, Gowthaman Govindarajan, Louise D. McCullough, Cintia S. de Paiva

**Affiliations:** 1 Ocular Surface Center, Cullen Eye Institute, Department of Ophthalmology, Baylor College of Medicine, Houston, TX 77030, USA; 2 Biochemistry and Cell Biology Graduate Program, Department of BioSciences, Rice University, Houston, TX 77005, USA; 3 BRAINS Research Laboratory, Department of Neurology, McGovern Medical School, The University of Texas Health Science Center at Houston, Houston, TX 77030, USA

**Keywords:** heterochronic parabiosis, aging, inflammation, inflammaging, lacrimal gland, dacryoadenitis

## Abstract

Aging is associated with inflammation and oxidative stress in the lacrimal gland (LG). We investigated if heterochronic parabiosis of mice could modulate age-related LG alterations. In both males and females, there were significant increases in total immune infiltration in isochronic aged LGs compared to that in isochronic young LGs. Male heterochronic young LGs were significantly more infiltrated compared to male isochronic young LGs. While both females and males had significant increases in inflammatory and B-cell-related transcripts in isochronic and heterochronic aged LGs compared to levels isochronic and heterochronic young LGs, females had a greater fold expression of some of these transcripts than males. Through flow cytometry, specific subsets of B cells were increased in the male heterochronic aged LGs compared to those in male isochronic aged LGs. Our results indicate that serum soluble factors from young mice were not enough to reverse inflammation and infiltrating immune cells in aged tissues and that there were specific sex-related differences in parabiosis treatment. This suggests that age-related changes in the LG microenvironment/architecture participate in perpetuating inflammation, which is not reversible by exposure to youthful systemic factors. In contrast, male young heterochronic LGs were significantly worse than their isochronic counterparts, suggesting that aged soluble factors can enhance inflammation in the young host. Therapies that aim at improving cellular health may have a stronger impact on improving inflammation and cellular inflammation in LGs than parabiosis.

## 1. Introduction

Aging leads to an increased risk of the development of many pathologies, including the development of dry eye disease, an ocular surface disorder [1]. Dry eye disease includes the elevation of CD4^+^ T cells in the conjunctiva, higher ocular surface inflammation, a decline in conjunctival goblet cell density, and disruption of the corneal barrier [2]. Dry eye is more frequent in females than males, but both sexes are affected [3]. 

Aging affects both the structure and function of the lacrimal gland, the producer of the aqueous layer in the tear film [4,5]. These glands suffer many age-related alterations, such as atrophy, inflammation, and fibrosis [6]. We and others have previously reported an increase in infiltrating mononuclear cells and the disruption of healthy cellular components, such as acinar cells, in aged lacrimal glands [7,8,9]. These infiltrating mononuclear cells are dispersed through the parenchyma of the gland but in many cases accumulate, forming foci of lymphocytes that can be quantified in histological sections by calculating the focus score, a measurement of total gland infiltration [10]. The enhanced infiltration of lymphocytic cells, including CD4^+^ T cells, B cells, and CD4^+^Foxp3^+^ cells is seen with aging [2,4,11]. In addition, the aged lacrimal gland exhibits a decrease in peroxidase secretion and a reduction in afferent and efferent nerve function [4,5]. We have previously characterized the cytokine milieu of aged lacrimal glands and observed a significant increase in B-and-T-cell-related cytokines [12]. These include cytokines that have been implicated in lymphocyte influx [8], such as *Il1b* and *Tnf* as broad inflammatory markers and *Ciita* (Class II major histocompatibility complex transactivator) and cathepsin S (*Ctss*), which are involved in antigen-presentation and MHC processing [13,14,15,16]. Furthermore, interferon-γ, secreted by activated NK and CD4^+^ T cells, can cause glandular apoptosis [17,18,19]. B cells are a specialized type of lymphocyte that can be divided into subsets based on extracellular and intracellular markers and have many distinct functions. For example, marginal zone-like B (MZB) cells are innate lymphocytes that can mount T cell-independent responses [20]. There are several markers associated with B cells, including *Cxcl13* and *Cxcl9*, chemokines involved in B cell migration and germinal center formation [21,22].

Parabiosis is a method first developed in 1864 to study the effect of a shared circulatory system [23]. Since then, it has been modified to reduce pain and infection in the animals but always involves surgically joining two animals, allowing for the development of a shared microvasculature between them, creating a shared chimeric circulation [24]. From its invention, parabiosis has been used to identify soluble factors that impact diseases, such as cancer, diabetes, hypertension, and obesity, as well as biological phenomena, such as stem cell differentiation and tissue regeneration, and factors involved in the aging process [25]. In studies of aging, heterochronic parabiosis involves pairing two mice together of different ages, while isochronic parabiosis involves pairing mice of the same age [26].

Parabiotic studies have increased since their revival in 2005 when parabiosis was used to investigate the aging of somatic stem cells [27]. However, few studies have investigated the potential effects of parabiosis and shared circulation on the eye. Hamrah and colleagues used parabiosis to study the turnover rate of bone marrow-derived cells in the cornea [28], and Wieghofer and associates used parabiosis to map myeloid populations in compartments of the eye [29]. In the retina, Heuss et al. made use of parabiosis to study the contribution of circulating mononuclear cells to an optic nerve injury [30]. Finally, Li Rong used parabiosis to investigate the mechanism of retinal aging [31]. However, the effects of parabiosis on the aged lacrimal gland and its effect on age-related dry eye disease have not been studied.

The purpose of this work was to use heterochronic parabiosis for the first time to study age-related dry eye disease in the lacrimal gland. We wanted to determine the role of soluble serum factors in the development of pathologies associated with lacrimal gland aging. We hypothesized that aged mice in heterochronic pairings would have reduced inflammation and pathology due to beneficial soluble factors present in the young parabiont. To do this, we made use of young PepBoy mice that carry the pan leukocyte marker CD45.1 and aged B6 mice that carry the marker CD45.2, so that that cellular origin of the leukocyte populations could be determined in the analysis.

Our results indicated that while heterochronic parabiosis for a period of eight weeks was not able to reverse the immune infiltration and inflammation seen in the aged lacrimal gland, pairing led to an increase in immune cells found in the young lacrimal gland. Our findings suggest that the aged lacrimal gland cannot be rejuvenated with young soluble factors, but soluble factors or cells from aged mice can lead to phenotypic features of aging in the young lacrimal gland. The identification of these detrimental factors could lead to targeted approaches to reduce eye aging. 

## 2. Results

### 2.1. Lacrimal Gland Pathology Was Worsened in Heterochronic Young Mice

**Figure 1:**
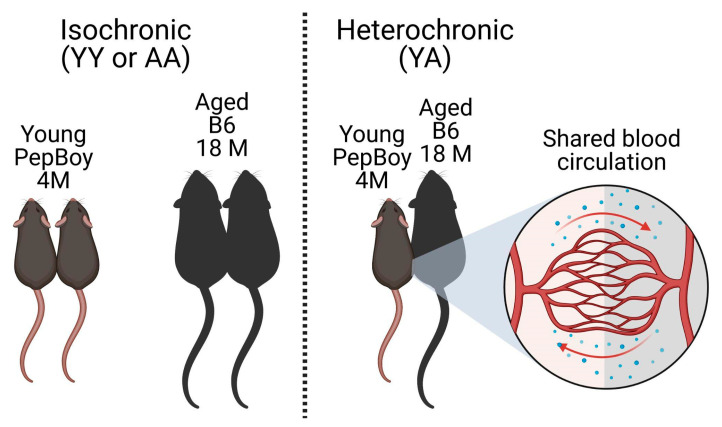
Schematic of parabiosis methodology. Mice were surgically joined at the thoracic and abdominal area, skin-to-skin, for 8 weeks before analysis. Isochronic pairings were either young–young (PepBoy to PepBoy) or aged–aged (B6 to B6). Heterochronic pairings were young–aged (PepBoy to B6). All ages listed were starting ages. Created with BioRender.com.

**Figure 2:**
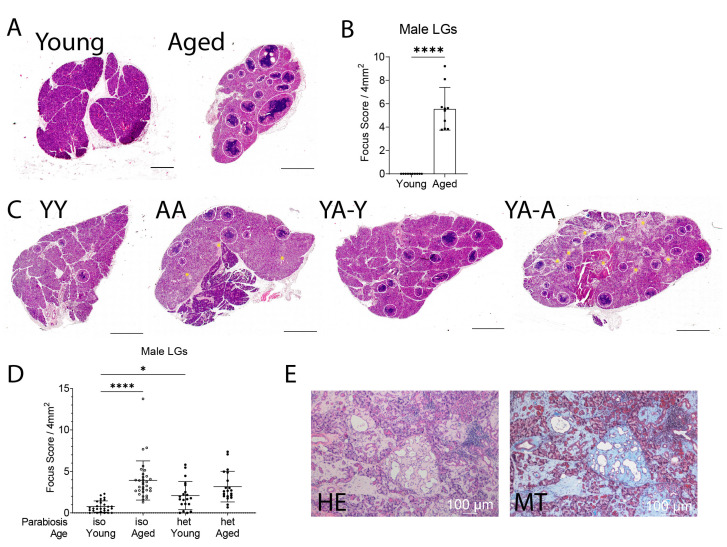
Heterochronic parabiosis worsens the pathology of the lacrimal gland in young heterochronic mice. (**A**) Representative H&E-stained scans of non-parabiotic lacrimal glands, young (PepBoy) and aged (B6). Each white circle is one focus. Scale bar is 1000 µm. (**B**) Representative focus score of non-parabiotic males. Each dot represents the average of two levels (as described in the Methods) per lacrimal gland per mouse. Mann–Whitney U test. **** = *p* < 0.0001. (**C**) Representative H&E-stained scans of parabiotic lacrimal glands. Each white circle is one focus. Yellow asterisks highlight areas of fibrosis. YY = isochronic young mice. AA = isochronic aged mice. YA-Y = young mouse in heterochronic young–aged pairing. YA-A = aged mouse in heterochronic young–aged pairing. Scale bar is 1000 µm. (**D**) Focus score calculated for parabiotic lacrimal glands. Each dot represents average of two levels per lacrimal gland per mouse. Non-parametric Kruskal–Wallis test followed by post hoc Dunn’s multiple comparisons test. * = *p* < 0.05; **** = *p* < 0.0001. (**E**) H&E section of male heterochronic aged mouse lacrimal gland compared to Masson’s trichrome (MT) of the same lacrimal gland. Blue represents collagen staining.

We first investigated the impact of 8-week-parabiosis on male lacrimal gland histology using heterochronic (young [6 months]/aged [20 months]) and isochronic (young/young or aged/aged) pairs (Figure 1). Mice were joined at the thoracic and abdominal area, skin-to-skin. Aged non-parabiotic C57BL/6 (B6; CD45.2) male mice have increased lymphocytic infiltration compared to young PepBoy male mice (CD45.1), which have none (Figure 2A,B). Similarly, isochronic aged male mice had increased lymphocytic infiltration compared to isochronic young male mice (Figure 2C,D). Surprisingly, young isochronic mice had focus scores greater than zero, which was not found in non-parabiotic mice (Figure 2B). Heterochronic young glands had significantly higher focal scores than isochronic young lacrimal glands (*p* = 0.047) and were not significantly different from heterochronic aged lacrimal gland focus scores (*p* = 0.2759).

A microscopic evaluation of HE-stained lacrimal glands showed that some aged male lacrimal glands (regardless of pairing) had areas of fibrosis that were not only periductal but also extended into the glandular parenchyma (Figure 2E). We confirmed the presence of fibrosis using Masson’s trichrome stain, indicating extensive collagen deposition (Figure 2E). We then compared the frequency of fibrosis in lacrimal glands between aged non-parabiotic and aged parabiotic mice and found that there was some amount of fibrosis regardless of parabiosis. Parabiotic aged lacrimal glands had about a 36% rate of fibrosis, while non-parabiotic aged mice had that of about 33%.

### 2.2. Significant Increase in Inflammatory and B-Cell-Related mRNA in Aged Lacrimal Glands Regardless of Parabiosis

**Figure 3:**
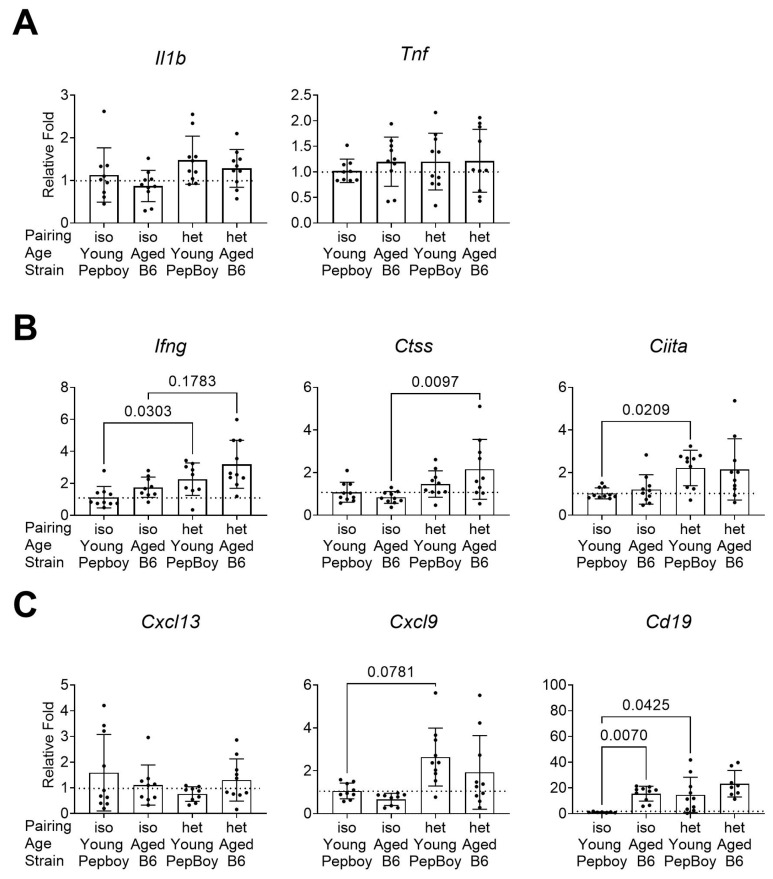
Gene expression analysis of lacrimal glands identifies B cell and inflammatory marker expression differences across age in the different parabiotic groups. (**A**) Broad inflammatory markers. (**B**) Antigen processing and presentation markers. (**C**) Chemokine and B cell markers. Each dot represents one lacrimal gland per mouse. Non-parametric Kruskal–Wallis followed by post hoc Dunn’s multiple comparisons test. iso = isochronic (young–young or aged–aged) pairing. het = heterochronic (young–aged) pairing.

Because we noted increased lymphocytic infiltration among the parabiotic groups, we next analyzed the fold expression of several inflammatory markers via qPCR. There was no difference in the expression of *Il1b* or *Tnf*, although the young heterochronic lacrimal gland had the highest levels of *Il1b* among the groups (Figure 3A). There was a significant increase in *Ifng* in the aged heterochronic mice compared to that in isochronic aged mice (Figure 3B). Consistent with the increased focus score in the young heterochronic compared to young isochronic groups, the young heterochronic gland showed elevated levels of *Ifng* and *Ciita*.

Because we observed an increase in lymphocytic infiltration in the heterochronic young lacrimal gland, we investigated if there was an increase in chemokines. While there were no differences in the expression levels of *Cxcl13*, there were significant fold increases in *Cxcl9*, a B-cell-attracting chemokine, in the young heterochronic gland (Figure 3C). Since there was an increase in the expression of a B-cell-related chemokine, we then investigated changes in *Cd19*, a gene marker for B cells. There was higher fold expression of *Cd19* in both the heterochronic young gland and the isochronic aged gland.

### 2.3. Increased T and B Cell Populations in Heterochronic Young Lacrimal Glands

**Figure 4:**
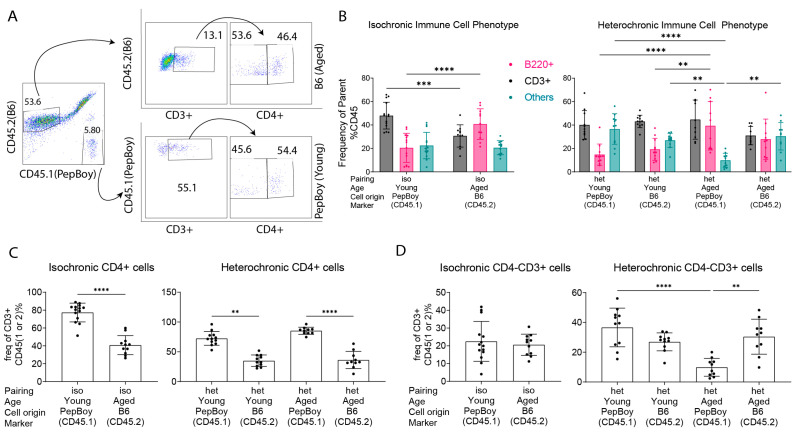
Flow cytometry analysis identifies changes in immune cell phenotypes and T cell subsets across groups. (**A**) Representative gating strategy of the lacrimal gland population from a heterochronic aged mouse stained with CD45.1, CD45.2, CD3, and B220 antibodies to identify major cell populations. CD45.1 or CD45.2 populations were identified and the frequency of CD3^+^, CD4^+^, and B220^+^ cells was calculated in each gate. (**B**) Cumulative data of immune cell phenotype quantification from isochronic and heterochronic mice in single cell lysates stained with CD45.1, CD45.2, CD3, and B220 antibodies. Each dot represents one sample. Two-way ANOVA followed by post-hoc Šídák multiple comparisons test. ** = *p* < 0.01; *** = *p* < 0.001; **** = *p* < 0.0001. (**C**) Cumulative data of CD4^+^CD3^+^ T cells from isochronic and heterochronic mice in single cell lysates stained with CD45.1, CD45.2, CD3, and CD4 antibodies. Each dot represents one sample. Non-parametric Kruskal–Wallis test followed by post-hoc Dunn’s multiple comparisons test. ** = *p* < 0.01; *** = *p* < 0.001; **** = *p* < 0.0001. (**D**) Cumulative data of CD4^−^CD3^+^ T cells from isochronic and heterochronic mice in single cell lysates stained with CD45.1, CD45.2, CD3, and CD4 antibodies. Each dot represents one sample. Non-parametric Kruskal–Wallis test followed by post-hoc Dunn’s multiple comparisons test. ** = *p* < 0.01; **** = *p* < 0.0001.

The results from the histologic evaluation in Figure 2 and Figure 3 suggest an influx of different immune cell types into the lacrimal gland. To better characterize these cells, we used flow cytometry on single cell lacrimal gland suspensions, taking advantage of the fact that PepBoy mice (young) can be identified with the CD45.1 marker while B6 mice (aged) can be identified with the marker CD45.2 (Figure 4A). Flow cytometry analysis confirmed chimerism of cells into the lacrimal gland: in heterochronic young mice, 35% of immune cells were from its B6 partner; in heterochronic aged mice, 25% of immune cells were from its PepBoy partner.

We first investigated the broad composition of the immune cells by using B, CD3, and CD4 antibodies. In isochronic pairings, young mice had a greater percentage of CD3^+^ T cells but a smaller percentage of B cells compared to those in isochronic aged mice (Figure 4B). In the heterochronic pairing, aged mice had a greater number of CD3^+^ cells from its young partner than from its own immune system, although this was not significant (*p* = 0.0622). These CD3^+^ cells were further divided into CD4^+^ and CD4^−^ subsets. The young isochronic gland had more CD4^+^ cells than the isochronic aged (*p* < 0.0001), and in the heterochronic pairing, there were significantly more CD4^+^ cells from the heterochronic young mouse in both glands (Figure 4C). In both heterochronic mice, there were significantly more CD4^−^ cells from its own system than from its partner. Heterochronic aged mice also had a greater increase in immune cells other than B220^+^ or CD3^+^ from its own system than from its young partner (*p* = 0.0013). Significantly, heterochronic aged mice had higher populations of B220^+^ cells, regardless of the source (Figure 4B).

**Figure 5:**
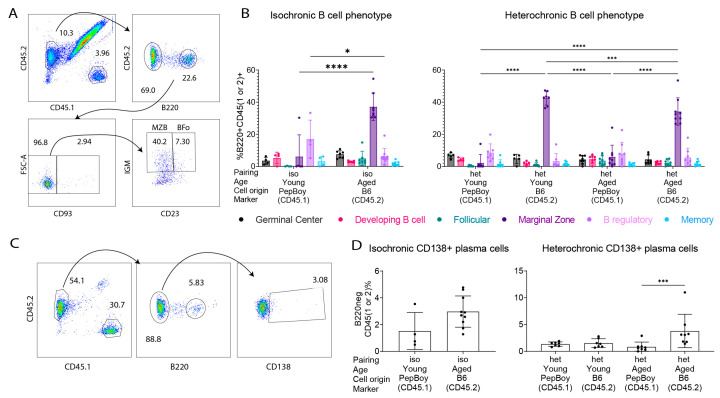
Flow cytometry analysis identifies changes in B cell populations across groups. (**A**) Representative gating strategy of the lacrimal gland population from a heterochronic aged mouse stained with CD45.1, CD45.2, B220, CD93, CD23, and IgM antibodies to identify MZB cells and B follicular-like cells (Bfo). CD45.1 and CD45.2 populations were identified, from which B220^+^ cells were found and used to identify CD93^−^ cells. MZB cells were labeled as CD93^−^CD23^−^IgM^+^, and Bfo cells were labeled as CD93^−^CD23^+^IgM^+^. (**B**) Cumulative data of B cell phenotype quantification in isochronic and heterochronic mice based on single cell lysates stained with CD45.1, CD45.2, B220, GL7, CD93, CD23, CD80, IgM, and IL-10 antibodies. Markers for each of the subsets were all B220^+^CD45.1 or CD45.2^+^ and the following: germinal center = GL7^+^; developing B cell = CD93^+^; follicular = CD93^−^CD23^+^IgM^+^; marginal zone = CD93^−^CD23^−^IgM^+^; B regulatory = IL-10^+^; memory = CD80^+^. iso = isochronic (young–young or aged–aged) pairing. het = heterochronic (young–aged) pairing. Two-way ANOVA followed by post-hoc Šídák multiple comparisons test. * = *p* < 0.05; *** = *p* < 0.001; **** = *p* < 0.0001. (**C**) Representative gating strategy of lacrimal gland population from a heterochronic aged mouse stained with CD45.1, CD45.2, B220, and CD138 antibodies to identify plasma cells. CD45.1 and CD45.2 populations were identified, followed by the B220^−^ population, and then CD138^+^ cells, which were identified as plasma cells. (**D**) Cumulative data of CD138^+^ plasma cell quantification from all parabiotic groups stained with CD45.1, CD45.2, B220, and CD138 antibodies. iso = isochronic (young–young or aged–aged) pairing. het = heterochronic (young–aged) pairing. Each dot represents one sample. Non-parametric Kruskal–Wallis test followed by post-hoc Dunn’s multiple comparisons test. *** = *p* < 0.001.

The results above indicated greater influx of B cells. To better characterize these B cells, we used B220 as a positive identifier of B cells for all subsequent subsets (Figure 5) using flow cytometry. Immune cells isolated from lacrimal glands were stained with CD45.1 or CD45.2 and then subsequently gated on B220, CD93, IgM, and CD23 expression. B220^+^CD93^+^ were defined as developing B cells. B220^+^CD93^−^ cells were further divided into MZB cells (CD93^−^CD23^−^IgM^+^) or follicular-like B cells (CD93^−^CD23^+^IgM^+^) depending on the expression of CD23 and IgM. In other panels, cells were stained with CD45.1, CD45.2, B220, and GL7 markers, the IL-10 marker, or with the CD80 marker to identify germinal center cells, B regulatory cells, and memory cells, respectively.

The results were therefore normalized as a percentage of B220^+^ cells. As we did in Figure 4B, cells were examined based on the expression of CD45.1 or CD45.2 to investigate the origin of the infiltrating B cells. MZB cells were the most abundant B220^+^ subtype among the subtypes (germinal center, developing B cell, follicular, memory, B regulatory, and marginal zone). They were enriched in isochronic aged mice compared to numbers in isochronic young mice (*p* < 0.001, Figure 5B). In heterochronic young mice, the highest proportion of MZB cells was from the aged (B6) partner (*p* < 0.0001), while in the heterochronic aged mouse, the highest proportion of MZB cells was from itself (*p* < 0.0001). There was a decrease in B regulatory cells (IL-10^+^B220^+^ cells) in isochronic aged mice compared to numbers in the isochronic young (*p* = 0.0184).

For the evaluation of plasma cells, single cell suspensions were stained with CD45.1, CD45.2, B220, and CD138 antibodies. B220^−^ cells were then gated based on the expression of CD138 (Figure 5C). Heterochronic aged mice had a greater proportion of plasma cells (B220^−^CD138^+^) from themselves than from their young partner (*p* = 0.0079), but these did not migrate to the heterochronic young gland (Figure 5D).

### 2.4. Heterochronic Parabiosis in Female Lacrimal Glands Does Not Improve Lymphocytic Infiltration or Inflammatory Marker Expression

**Figure 6:**
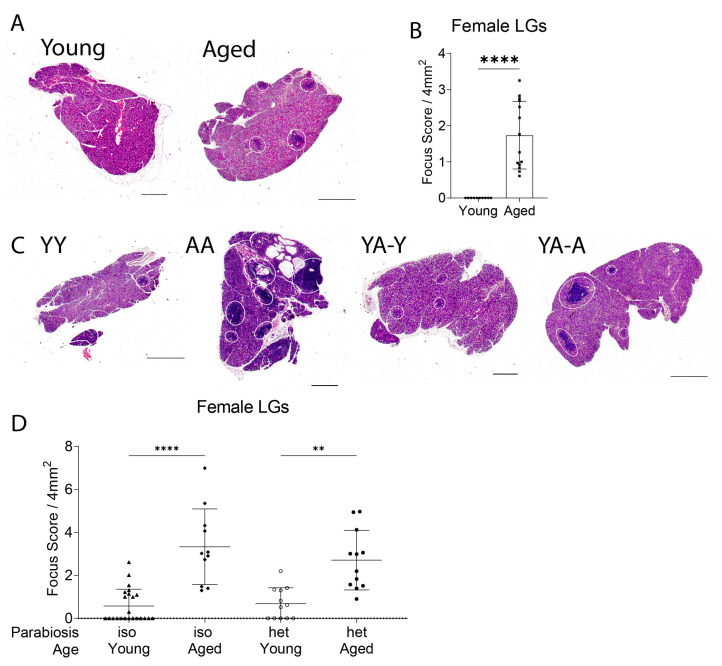
Female lacrimal gland infiltration followed the infiltration phenotype seen in aging, but that in heterochronic pairings was not worse than that in isochronic pairs. (**A**) Representative H&E-stained scans of female individual lacrimal glands, young (PepBoy) and aged (B6). Scale bar is 1000 µm. (**B**) Representative focus scores of non-parabiotic lacrimal glands. Each dot represents the average of two levels per lacrimal gland per mouse. Mann–Whitney U test. **** = *p* < 0.0001. (**C**) Representative H&E-stained scans of female parabiotic lacrimal glands. Each white circle is one focus. YY = isochronic young mice. AA = isochronic aged mice. YA-Y = young mouse in heterochronic young–aged pairing. YA-A = aged mouse in heterochronic young–aged pairing. Scale bar is 1000 µm. (**D**) Focus score calculated for female parabiotic lacrimal glands. Each dot represents the average of two levels per lacrimal gland per mouse. Non-parametric Kruskal–Wallis test followed by post-hoc Dunn’s multiple comparisons test. ** = *p* < 0.01; **** = *p* < 0.0001.

To rule out a potential bias in male sex regarding the effects of parabiosis, we added a cohort of female parabionts for analysis. Young female PepBoy and aged female B6 mice were surgically joined as described in the methods and in Figure 1. As with males, female parabiotic mice were analyzed after 8 weeks of parabiosis. First, we analyzed lacrimal gland pathology in histologic sections (Figure 6). Non-parabiotic young PepBoy females had no immune infiltration (focal score = 0), which significantly increased with age (*p* = 0.0007, Figure 6A,B). Isochronic aged female mice had significantly greater focal scores than isochronic young female mice (Figure 6D, *p* < 0.0001). Heterochronic aged female mice had significantly higher focal scores than their heterochronic young partners (*p* = 0.0047). However, unlike in males, based on a Kruskal–Wallis test followed by post-hoc Dunn’s multiple comparisons test, there were no statistical differences in focus scores between isochronic and heterochronic young females or isochronic and heterochronic aged females.

**Figure 7:**
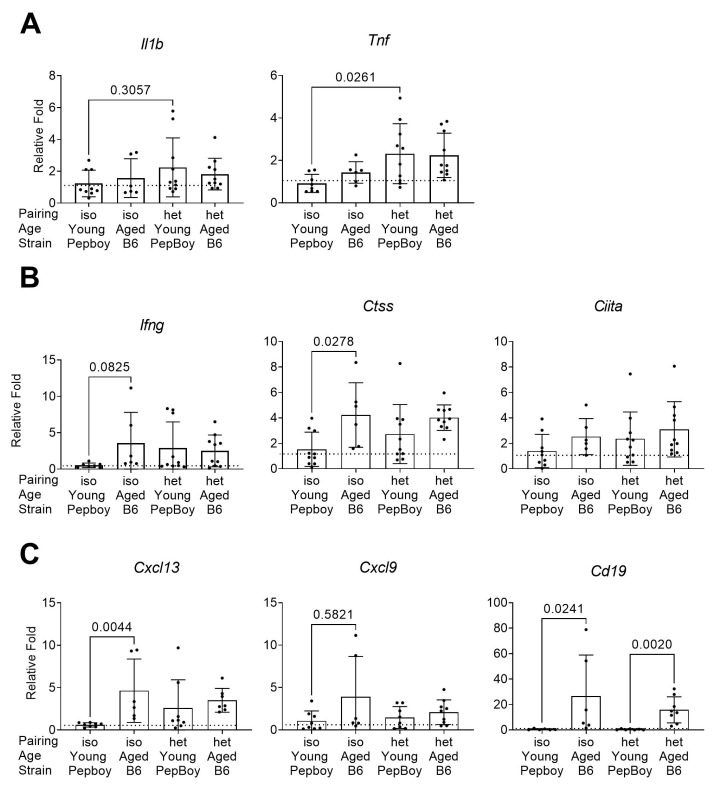
Gene expression analysis of B cell and inflammatory markers in female lacrimal glands has similar patterns of fold expression to those in males. (**A**) Broad inflammatory markers. (**B**) Antigen processing and presentation markers. (**C**) Chemokine and B cell markers. Each dot represents one lacrimal gland per mouse. Non-parametric Kruskal–Wallis test followed by post-hoc Dunn’s multiple comparisons test. iso = isochronic (young–young or aged–aged) pairing. het = heterochronic (young–aged) pairing.

Next, we investigated the same set of genetic markers in the female parabiotic lacrimal gland as we did with male parabiotic glands via qPCR. While there was no change in the broad inflammatory marker *Il1b,* there was a significant increase in *Tnf* in the heterochronic young mice compared to levels in young isochronic glands (Figure 7A). There was increased expression of *Ctss* in the isochronic aged females compared to that in isochronic young females (Figure 7B). Likewise, *Ifng* showed increased expression in isochronic aged glands compared to that in the isochronic young group. *Ciita* levels were similar across all four groups. When we investigated markers for B-cell-related chemokines and B cells, we found a significant increase in *Cxcl13* and *Cd19* in the isochronic aged females compared to levels in isochronic young females (Figure 7C). Interestingly, there was also significant *Cd19* expression in the heterochronic aged lacrimal gland compared to that in its heterochronic young partner, suggesting an increase in B cells in the heterochronic young lacrimal gland that mimicked isochronic aged glands.

### 2.5. Aged Male Mice Have Worse Lacrimal Gland Infiltration and a Greater Frequency of Fibrosis than Aged Female Mice

To account for sex-specific differences, we compared our results using sex as a biological variable. The differences between male and female lacrimal gland pathology and the genetic expression of inflammatory markers are summarized in Table 1 and in Supplemental Figures S1 and S2. Lacrimal gland total infiltration scores followed similar trends across isochronic aged groups regardless of sex, but young male heterochronic glands had greater focal scores than those of females in the same group (*p* = 0.0598). Aged male lacrimal glands also had fibrosis, regardless of pairing, while aged female lacrimal glands did not (Supplemental Figure S1C).

Certain genetic markers were also more broadly expressed in one sex over the other. *Tnf* and *Ctss* were expressed at higher levels in the heterochronic aged female mice compared to those in heterochronic aged male mice (Table 1, Supplemental Figure S2). *Ctss* and *Cxcl9* were expressed at higher levels in the isochronic aged female mice than in isochronic aged male mice. Only *Cxcl13* was expressed higher in male mice, in isochronic young pairings.

In summary, while male mice appeared to have a worse phenotype, based on histology, than female mice, in general, female mice had greater fold expression of inflammatory and B-cell-related genetic markers than male lacrimal glands.

## 3. Discussion

Aging is a risk factor for many inflammatory pathologies, including dry eye disease [3,32]. The tear-secreting lacrimal gland contributes the aqueous portion of the tear film [33]. In dry eye disease, its function can be severely disrupted due to the infiltration of lymphocytes, resulting in less tear secretion [34,35]. Likewise, aging results in significant changes to the lacrimal gland, including the loss of function, acinar atrophy, periductal fibrosis, lymphocytic infiltration, and ductal dilation [6,8,36,37,38,39]. It is therefore important to find therapies that can address alterations to the lacrimal gland that occur during aging to increase the lifespan of the gland. In this study, we investigated the use of heterochronic parabiosis as a treatment for age-related lacrimal gland inflammation, since the exchange of blood soluble factors has shown promising results in animal models of cognitive impairment [40] and stem cell rejuvenation [41]. While we hypothesized that heterochronic parabiosis would improve age-related changes in the lacrimal gland, instead we found that the aged gland’s soluble factors and environment eclipsed those of the young glands and caused phenotypic signs of aging and worsened lacrimal gland inflammation (dacryoadenitis) in young parabionts.

Therefore, the new findings of our study are that heterochronic parabiosis heightens an aged phenotype in young male lacrimal gland pathology, increases the expression of inflammatory and B-cell-related cytokine transcripts, and results in larger populations of B cells found in the young heterochronic lacrimal gland. To our knowledge, this is the first study investigating the use of an aging heterochronic parabiosis model in studies of age-related lacrimal gland infiltration and inflammation.

### 3.1. Lacrimal Gland Phenotype

A surprising and unexpected finding in our results was that the histology of the lacrimal gland was worsened in heterochronic young recipients. While the parabiosis procedure resulted in a greater increase in inflammation, isochronic young male lacrimal glands had less lymphocytic infiltration than heterochronic young male lacrimal glands. Indeed, young male heterochronic lacrimal gland focal scores were not significantly different from those of their heterochronic aged male counterparts, indicating that shared blood circulation with aged mice worsens the cellular environment of the young lacrimal gland, resulting in significantly worse lymphocytic infiltration. Our results agree with other studies showing deleterious effects of heterochronic parabiosis on young recipients. Jeon and colleagues reported an increase in cell and tissue senescence after one transfusion of aged blood to young mice [42]. They identified increased markers of kidney damage, liver fibrosis, and the senescence-associated secretory phenotype. Similarly, Pálovics and colleagues showed that many young tissues, when exposed to aged blood, took on an aging phenotype [43], which agreed with prior work [44]. In aged mice, the aging phenotype was only partially reversed in specific tissues exposed to young blood, most notably the liver, pancreas, and tissues that rely heavily on the mitochondrial electron transport chain. In the brain, heterochronic parabiosis resulted in positive changes for the aged recipients, but negative changes in the young recipients, largely due to activation of cell senescence pathways [45], impairment of neurogenesis [46], and inhibition of cognitive function [47]. Yankova et al. reported that heterochronic parabiosis resulted in a significantly shorter lifespan for young heterochronic recipients compared to that in isochronic young mice [48]. While we did not see an improvement in the heterochronic aged gland after eight weeks of parabiosis, we did find that aged factors from the aged lacrimal gland accelerated the aging phenotype in the young lacrimal gland. Identifying these factors that drive the aging phenotype in young glands could help to identify novel targets to help prevent age-related inflammation and destruction to the eye and lacrimal gland.

### 3.2. Inflammatory Marker Expression

Heterochronic parabiosis resulted in a higher fold expression of certain inflammatory and B-cell-related markers in the lacrimal gland, notably *Ifng*, *Ctss*, *Ciita*, and *Cxcl9.* As aging is accompanied by increased heterogeneity and variability [49,50], we observed a large variability in gene expression in our samples. We had previously shown an increase in *Ifng*, *Ctss*, and *Ciita* in the female aged lacrimal gland [8]. Interferon-γ has been shown to be a key player in aging and in dry eye. It exacerbates conjunctival apoptosis [19], results in goblet cell loss [51], and is secreted by infiltrating natural killer cells and CD4^+^ T cells to the conjunctiva [18], and its inhibition improves dry eye disease by increasing conjunctival goblet cells [52,53] and preventing lacrimal gland decline and destruction [54]. The increase in this mRNA transcript in both heterochronic recipients indicates an overall increase in inflammation and could be a main reason for the severity of lacrimal gland infiltration in the young heterochronic lacrimal gland. CXCL9 is a Th1-associated chemokine that helps coordinate the migration of Th1 cells. It requires interferon-γ for induction in dry eye disease [55]. Higher expression of *Cxcl9* could have led to the greater infiltration of lymphocytes to the lacrimal gland, as was seen with their higher focal score (Figure 2C). Cathepsin S degrades the invariant Ii peptide in MHC II receptors for antigen docking and eventual presentation [56]. It has been implicated in several autoimmune diseases, including autoimmune myasthenia gravis pathogenesis [57], systemic lupus erythematosus [58], diabetes [59], and Sjögren Syndrome, a prototype autoimmune dry eye disease [60,61]. We have reported previously that *Ctss* mRNA is increased in aged murine female lacrimal glands [62] and that there is increased activity of the protein cathepsin S in both the tears of patients with dry eye disease and aged mice [60,62]. Aged *Ctss*^−/−^ mice had improved corneal barrier functions and goblet cell density (both hallmarks of dry eye) compared to aged wild-type mice [62]. It is notable that *Ctss* mRNA expression was increased in heterochronic aged mice compared to that in isochronic aged mice, indicating that a marker for dry eye disease was heightened in the heterochronic parabionts. *Ciita* regulates MHC II expression [15]. Because of its main role in governing antigen-presenting cells, its presence strongly suggests that antigen-presenting cells are a key player in the pathology of the lacrimal gland. These findings suggest that while heterochronic parabiosis did not improve the pathology of aged lacrimal glands, it caused young lacrimal glands to develop dacryoadenitis, indicating that the exchange of soluble factors with young mice worsened the immune cell response in aged parabiotic glands.

### 3.3. Immune Cell Identification

The heterochronic aged lacrimal gland had increased proportions of specific B cell subsets and more T cells than its isochronic counterpart. Most T cells came from the young partner, which suggests that the young mouse provided active CD4^+^ T cells that circulated throughout both glands. On the other hand, the proportion of B cells from itself and its partner were not significantly different. This suggests that the inflamed microenvironment of the lacrimal gland attracted immune cells indiscriminately and perpetuated inflammation, indicating that the aged microenvironment was a stronger factor than the regulation of immune cells when it came to autoimmune destruction of the tissue. In isochronic aged mice, there was a significant decrease in B regulatory cells compared to levels in isochronic young mice, which might indicate that some of the autoimmune changes seen in the isochronic aged mice were due to a lack of these B regulatory cells. However, the proportions of B regulatory cells did not significantly change in the heterochronic mice, suggesting that other factors are also at play. We have shown that immune cells in the aged lacrimal glands develop ectopic lymphoid structures (or tertiary lymphoid tissue) that have high levels of germinal centers and T follicular helper cells [12]. Here, it is possible that these ectopic lymphoid structures drew circulating immune cells from both recipients to reside in the lacrimal gland. The most important B cell subset was MZB cells, which were significantly increased in isochronic aged mice compared to numbers in the isochronic young group. In the heterochronic young lacrimal gland, MZB cells from its aged partner were significantly more abundant than MZB cells from its own circulation, demonstrating that aged MZB cells are very sensitive to the chemoattractant gradient. We also found that MZB cells accumulate in non-parabiotic lacrimal glands, in contrast to B follicular cells, which accumulate in the draining lymph nodes [12]. MZB cells produce antibodies during infection [20]. The increases seen in both the heterochronic and isochronic lacrimal glands here suggest dysregulation of the immune system associated with aging [63,64,65,66], resulting in higher levels of autoreactive immune cells, including MZB cells. This could also be a reason for the greater infiltration of lymphocytes in the heterochronic young lacrimal gland. Taken together, these results indicate that heterochronic parabiosis results in the circulation of active T cells from the young partner and mostly MZB cells from the aged partner to both lacrimal glands. This resulted in the worse focal scores for the heterochronic young glands (Figure 2C) and is potentially why the heterochronic aged focal scores did not improve when compared to the isochronic aged scores.

### 3.4. Sex Differences in Parabiotic Lacrimal Glands

One limitation of this study was the limited availability of aged female mice for analysis; therefore, we restricted our analysis in females to the focus score and gene expression. On a histological level, heterochronic young lacrimal glands were not as severely infiltrated as their male counterparts, suggesting that female parabiosis neither improved the aged mice nor worsened the young in the heterochronic pairs. However, female lacrimal glands exhibited higher fold expression of several inflammatory markers, including *Tnf*, *Ctss*, *Cxcl9*, and *Cd19*, suggesting a greater number of B cells infiltrating these glands. This might suggest that while there were fewer foci in the gland, there was an increased presence of diffuse lymphocytes to the tissue.

Both heterochronic and isochronic aged male lacrimal glands had a high prevalence of fibrosis, which was not seen in young lacrimal glands. This finding was not seen in the female lacrimal glands, indicating a sex-specific difference in the aging lacrimal gland. Work investigating the role of sex hormones in the lacrimal gland also reported significant sex differences in aging due to the effects of estrogen signaling in the male lacrimal gland [67]. When we compared the rate of fibrosis between age-matched parabiotic and non-parabiotic mice, there was no difference in frequency. We also investigated a set of diversity outbred lacrimal glands in our recent publication [12] to see if the incidence of fibrosis was specific to C57BL/6J mice and found fibrosis in the aged males, but not the aged females. Other work has identified sex differences in genetic marker expression in the lacrimal gland, finding significant sex changes in inflammatory markers, such as CXCL9 and CXCL13 [68]. Although we did not find fibrosis in the young heterochronic lacrimal gland, heterochronic parabiosis has resulted in liver fibrosis in young recipients [42,44]. Investigating the sex-specific fibrosis found in the lacrimal glands may prove to be an interesting avenue of further research.

Our findings indicate that age-related pathologies in male and female lacrimal glands are different processes. Male mice had increased lymphocytic infiltration because of parabiosis, while female mice had increased expression of several inflammatory cytokines. These data suggest that female and male aged mice responded differently to parabiosis, but aging resulted in an increase in inflammation in both sexes that was not improved with heterochronic parabiosis.

## 4. Materials and Methods

All experiments were approved prior to their execution by the University of Texas Health Science Center and Baylor College of Medicine’s Institutional Animal Care and Use Committees. The studies also followed all guidelines for the Use of Animals in Ophthalmic and Vision Research as supported by the Association for Research in Vision and Ophthalmology and the NIH Guide for the Care and Use of Laboratory Animals (NIH Publications No. 8023, revised 1978 [69]). The Ocular Surface Center, Department of Ophthalmology, Baylor College of Medicine (Houston, TX, USA) and the BRAINS Research Laboratory, Department of Neurology, The University of Texas Health Science Center at Houston John P and Katherine G McGovern Medical School (Houston, TX, USA) were where all experiments took place.

### 4.1. Animals

Male and female PepBoy mice 4 months of age were surgically joined to either another PepBoy or a C57BL/6J (B6) mouse aged 18 months at the thoracic and abdominal area, skin-to-skin. The peritoneum remained intact. Pairings were either isochronic (young to young or aged to aged) or heterochronic (young to aged), all same-sexed. Male mice were used in the following numbers: isochronic young (YY) = 40 pairs, isochronic aged (AA) = 35 pairs, heterochronic young (YA-Y) = 36 mice, heterochronic aged (YA-A) = 36 mice, non-parabiotic 4-month PepBoy = 10 mice, non-parabiotic 19-month B6 = 10 mice. Female mice were used in the following numbers: YY = 12 pairs, AA = 14 pairs, YA-Y = 13 mice, YA-A = 13 mice, non-parabiotic 4-month PepBoy = 10 mice, non-parabiotic 21 month B6 = 14 mice. Mice were evaluated 2 months post-surgery. Surgeries were performed at the BRAINS Research Laboratory in the University of Texas Health Science Center at Houston John P and Katherine G McGovern Medical School. After euthanasia, tissues were shared. Extra-orbital lacrimal glands were collected and processed either for histology, gene expression analysis, or flow cytometry.

### 4.2. Calculation of Lymphocytic Infiltration

Lacrimal glands (male, number of glands: YY = 29, AA = 32, YA-Y = 20, YA-A = 20, non-parabiotic young PepBoy = 10, non-parabiotic old B6 = 10; female, number of glands: YY = 24, AA = 11, YA-Y = 13, YA-A = 12, non-parabiotic young PepBoy = 10, non-parabiotic old B6 = 14) were excised, fixed in 10% formalin, paraffin-embedded, and cut into 4 µm sections using a microtome (ThermoFisher, Microm HM 315, Waltham, MA, USA). Sections were stained with H&E using an autostainer (Gemeni AS, ThermoFisher, Waltham, MA, USA), and coverslips were applied (Tissue Tek Auto Cover Slipper 4764, Sakura, Torrance, CA, USA). These procedures were performed at Precise Pathology Associates, PLLC (The Woodlands, TX, USA). Lacrimal glands were cut into 5 different levels and each staining level was 12 µm apart from the next one. Using a standard light microscope with a 10X objective (Nikon, Tokyo, Japan; Eclipse E400), two blinded observers, to prevent bias, counted lymphocytic infiltrate foci. A minimum of 50 mononuclear cells was counted as one focus. Slides were scanned with a PathScan Enabler V (Meyer Instruments, Houston, TX, USA) and were calibrated according to the manufacturer’s instructions (2.54 µm/pixel) using NIS Elements software (version 5.30.05). The total area of the scanned lacrimal gland was calculated using NIS Elements. The focus score was calculated as the total number of counted foci on a scanned lacrimal gland per 4 mm^2^, to match human pathologist standards [10].

### 4.3. Masson’s Trichrome Stain

Lacrimal glands (male, number of glands: AA = 4, YA-A = 4) were excised, fixed in 10% formalin, paraffin-embedded, and cut into 5 µM sections using a microtome (Microm HM 340E; Thermo Fisher Scientific, Waltham, MA, USA). Masson’s trichrome stain kit (StatLab, McKinney, TX, USA) was used following the manufacturer’s protocol. Using a standard light microscope with a 10X objective (Nikon; Eclipse E400), two masked observers counted sections for fibrosis in lacrimal gland sections (absence = 0; presence = 1). Frequency was analyzed using Chi square analysis in GraphPad 9.1.

### 4.4. RNA Isolation and Real-Time PCR

We extracted RNA from excised extraorbital lacrimal glands (male, number of glands: YY = 10, AA = 10, YA-Y = 10, YA-A = 10; female, number of glands: YY = 10, AA = 6, YA-Y = 10, YA-A = 10) according to the manufacturer’s protocol, using a QIAGEN RNeasy Plus Mini RNA isolation kit (Qiagen, Hilden, Germany). To generate cDNA, we measured the concentration of RNA, calculated 1 µg of RNA for use, and then used the Ready-To-Go™ You-Prime First-Strand kit (GE Healthcare, Chicago, IL, USA). All real-time PCR used minor groove-binding Taqman probes, which were IFN-γ (*Ifng*, Mm01168134), major histocompatibility complex class II (*Ciita*, Mm00482914), TNF-α (*Tnf*, Mm00443258), IL-1β (*Il1b*, Mm00434228), Cathepsin S (*Ctss*, Mm00457902), CXCL13 (*Cxcl13*, Mm00444533), CXCL9 (*Cxcl9*, Mm00434946), and CD19 (*Cd19*, Mm00515420). Real-time PCR reactions used TaqMan™ Fast Universal PCR Master Mix (2X) and no AmpErase™ UNG (Thermo Fisher Scientific) and were all carried out on a Taqman Universal PCR system (StepOnePlus™ Real-Time PCR System, Applied Biosystems, Bedford, MA, USA). A housekeeping gene, hypoxanthine phosphoribosyltransferase 1 (HPRT1, Mm00446968), was used to normalize all Ct values.

### 4.5. Flow Cytometry

Lacrimal glands (male, number of glands: YY = 18, AA = 21, YA-Y = 19, YA-A = 18) were excised and incubated with 0.1% collagenase IV. Cell suspensions were frozen in 1 mL of freezing composed of 90% FBS (Gibco/ThermoFisher, Waltham, MA, USA) and 10% DMSO (ThermoFisher, Waltham, MA, USA) and stored in liquid nitrogen until analysis could be performed. Samples were thawed in a water bath and added to 9 mL of warmed RPMI media (ThermoFisher), before proceeding with analysis. Single-cell suspensions were incubated with anti-CD16/32 (BioLegend, San Diego, CA, USA) to block Fc receptors for 10 min at 4 °C and subsequently stained with one of three cocktails containing some of the following antibodies: anti-CD45.1_PE (Clone A20, BioLegend), anti-CD45.2_BV510 (Clone 104, BioLegend), anti-CD4_FITC (Clone RM4-5, Invitrogen/ThermoFisher, Waltham, MA, USA), anti-CD3_BB700 (Clone 145-2C11, BD BioSciences, Franklin Lakes, NJ, USA), anti-B220_APC (Clone RA3-6B2, BD BioSciences), anti-IgA_FITC (Clone MA-6E1, Invitrogen/ThermoFisher), anti-CD80_PerCpCy5.5 (Clone 16-10A1, BioLegend), anti-IL-10_PE (Clone JES5-16E3, BioLegend), anti-CD138_BV421 (Clone 281-2, BioLegend), anti-IgM_FITC (Clone RMM-1, BioLegend), anti-GL7_PerCpCy5.5 (Clone GL7, BioLegend), and anti-CD23_BV421 (Clone B3B4, BioLegend). Afterwards, cells were incubated with an infrared fluorescent reactive live/dead dye diluted 1:32 (ThermoFisher) for 20 min, washed, and resuspended in 2% formaldehyde for 15 min. Gates were determined by first finding cells on the forward scatter area versus side scatter area, and then finding singlets based on first forward scatter height versus forward scatter area, followed by a second doublet discrimination of side scatter height versus side scatter area. Alive cells were discriminated from dead using fixable infrared dye versus side scatter, and then, CD45.1^+^ cells and CD45.2^+^ cells were gated for subsequent analysis. CD3^+^CD4^+^ or B220^+^ cells were found to calculate the frequency of parents for combinations of subsets.

Negative controls consisted of fluorescence minus one (FMO) combined cell suspensions from all animal groups. Cells were acquired with the BD Canto II Benchtop cytometer with BD Diva software version 6.7 (BD Biosciences). Final data were analyzed using FlowJo software version 10 (Tree Star, Inc., Ashland, OR, USA).

### 4.6. Statistical Analysis

We used GraphPad Prism (GraphPad Software, San Diego, CA, USA, version 9.1) to perform all statistical analyses. The Kolmogorov–Smirnov normality test was first used. Then, non-parametric (Mann–Whitney) statistical tests were used to make comparisons between two groups. When possible, we used one-way or two-way ANOVA or Kruskal–Wallis followed by post-hoc tests. *p* < or equal to 0.05 was considered significant.

## 5. Conclusions

Altogether, our results indicate that young blood shared with aged recipients does not reduce the pathology of age-associated dacryoadenitis. The aged microenvironment attracts immune cells that perpetuate tissue destruction and inflammation, and soluble factors from this environment go on to increase the aging phenotype and inflammation in the young lacrimal gland. Aged blood had a deleterious effect on the young lacrimal gland, resulting in an increase in lymphocytic infiltration, greater expression of inflammatory transcripts, and an increase in MZB cells. Identification of the factors that accelerated this aging phenotype could provide a novel avenue for therapeutics related to dry eye disease. Furthermore, there were differences in sex across experimental groups. Heterochronic parabiosis in female lacrimal glands had higher inflammatory cytokine expression but less foci infiltration than male lacrimal glands. The difference based on sex underscores a greater need for investigation into sex differences in aging and in response to parabiosis. Treatments for age-related dry eye should pursue addressing the inflamed microenvironment of the lacrimal gland and aged soluble factors to help stop this self-perpetuating cycle.

## Figures and Tables

**Table 1:** Sex differences found in lacrimal gland analyses. Two-way ANOVA followed by post-hoc Šídák multiple comparisons test. Only significant changes in qPCR results are shown. ↑↑↑ = prevalence of fibrosis. ╳ = no fibrosis.

Parameters	Sex Comparison	Male	Female	Sex Effect	*p*-Value
Lacrimal gland evaluation	Isochronic young focus score	0.76	0.57	M = F	*p* > 0.99
	Isochronic aged focus score	3.91	3.33	M = F	*p* = 0.92
	Heterochronic young focus score	2.1	0.68	M > F	*p* = 0.06
	Heterochronic aged focus score	3.16	2.71	M = F	*p* = 0.97
	Occurrence of fibrosis in aged lacrimal gland	↑↑↑	╳	M >> F	*p* < 0.001
Gene expression analysis in lacrimal gland	*Tnf* in heterochronic aged mice	1.22 fold	2.34 fold	F > M	*p* = 0.043
	*Ctss* in isochronic aged mice	2.26 fold	4.33 fold	F > M	*p* = 0.046
	*Ctss* in heterochronic aged mice	2.15 fold	4.02 fold	F > M	*p* = 0.024
	*Cxcl13* in isochronic young mice	8.24 fold	0.61 fold	M > F	*p* = 0.0003
	*Cxcl9* in isochronic aged mice	1.18 fold	3.90 fold	F > M	*p* = 0.024

## Data Availability

Data are contained within the article or supplementary material. Further inquiries can be directed to the corresponding author.

## Supplementary Materials

The following supporting information can be downloaded at: https://www.mdpi.com/article/10.3390/ijms24054897/s1.
