# Supplementary material for: Heterochronic Parabiosis Causes Dacryoadenitis in Young Lacrimal Glands
Source: Int J Mol Sci. 2023 Mar 3;24(5):4897. doi: 10.3390/ijms24054897 (PMC10003158; doi:10.3390/ijms24054897)
Supplement: Supplementary file 1 [file ijms-24-04897-s001.zip › ijms-2217096-supplementary.pdf]

**Supplemental Figures**

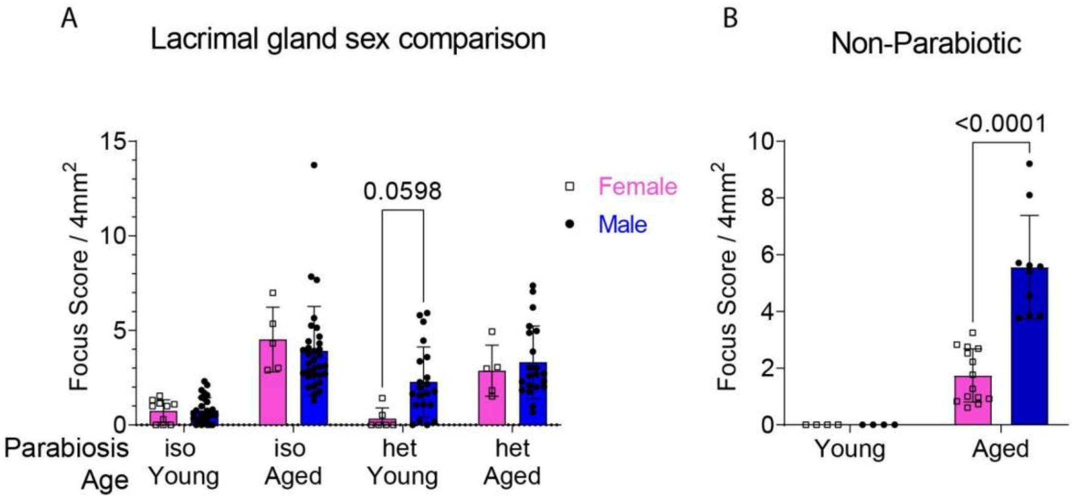


C

| | *Fibrosis* | *No Fibrosis* | *Total* | *p-value* |
|---|---|---|---|---|
| *Females* | 0 | 56 | 56 | NS |
| *Males* | 32 | 55 | 87 | <0.0001 |

***Supplemental Figure S1: Sex differences in lacrimal glands in both parabiotic and non-parabiotic models.*** *A) Sex differences in parabiotic lacrimal glands. iso = isochronic pairing (young to young or aged to aged). het = heterochronic pairing (young to aged). B) Focus score for non-parabiotic mice. Young are PepBoy, aged are B6. Pink bars indicate female mice, blue bars indicate male mice. Each dot represents average of two levels per lacrimal gland per mouse. Two-way ANOVA followed by post hoc Šídák multiple comparisons test. C) Chi-square analysis of frequency of fibrosis in aged lacrimal glands. Glands were pooled from non-parabiotic and parabiotic grading. Fisher's exact test.*

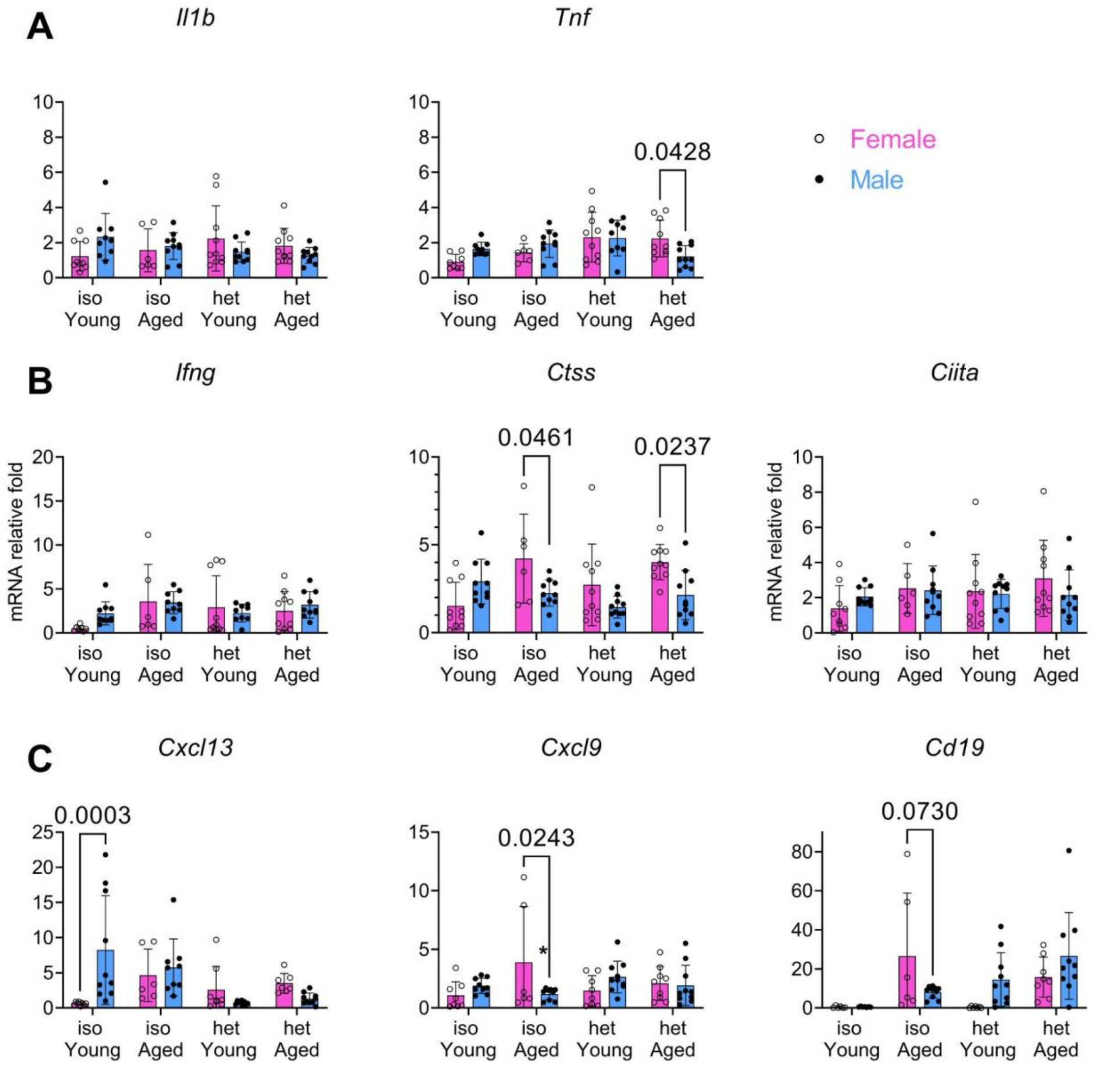


***Supplemental Figure S2: Sex differences in lacrimal gland fold expression of inflammatory and B cell related markers.*** *A) Broad inflammatory markers. B) T cell related markers. C) B cell related markers. Each dot represents one lacrimal gland per mouse. Pink bars indicate female mice, blue bars indicate male mice. Two-way ANOVA followed by post hoc Šídák multiple comparisons test. iso = isochronic (young-young or aged-aged) pairing. het = heterochronic (young-aged) pairing.*
